# Prevalence of sarcopenia in elderly psychiatric inpatients of central Slovenia - study protocol

**DOI:** 10.1192/j.eurpsy.2025.1757

**Published:** 2025-08-26

**Authors:** A. Stojkovska, P. Rus Prelog

**Affiliations:** 1 University Psychiatric Clinic; 2 Faculty of Medicine, University of Ljubljana, Ljubljana, Slovenia

## Abstract

**Introduction:**

Sarcopenia is a syndrome described as generalized and progressive loss of muscle mass, strength and function, leading to an increased risk of falls, fractures, disability and mortality. Recent studies have shown that sarcopenia is more common in patients with long-standing psychiatric illnesses compared to the general population, particularly in those with depression and dementia. There have also been reports of higher prevalence in patients with schizophrenia and bipolar disorder.

**Objectives:**

Our study aims to determine the prevalence of sarcopenia and the factors associated with the syndrome among the geriatric psychiatric population diagnosed with Alzheimer’s disease, depression, bipolar disorder, or schizophrenia in the central Slovenian region.

**Methods:**

A single-centre cross-sectional study will be conducted over 3 months at the Department of Geriatric Psychiatry of the University Psychiatric Clinic Ljubljana. Admitted patients aged 65 years or older with a diagnosis of Alzheimer’s, depression, bipolar disorder, or schizophrenia will be eligible (50 patients per each diagnosis). The SARC-F (Strength, Assistance with walking, Rising from a chair, Climbing stairs, and Falls) and the MSRA (Mini Sarcopenia Risk Assessment) questionnaires will be used as screening tools to identify sarcopenic patients. Additionally, calf circumference and handgrip strength will be measured. The patients’ cognitive functioning will be assessed using the Mini‐Mental State Evaluation (MMSE).

**Results:**

We expect to detect higher rates of sarcopenia among inpatients with mental illness. Subgroups will be analyzed according to different demographic and disease parameters.

**Conclusions:**

This study is the first to evaluate sarcopenia among elderly Slovenian inpatients with a mental health illness. We believe that sarcopenia is an important yet underdiagnosed parameter of functional health in the elderly struggling with a chronic mental health illness and should be regularly screened for. Further research is required to identify sarcopenia in this vulnerable population, especially at an early stage, to counteract the progression of the disease.

**Disclosure of Interest:**

None Declared

